# Quality of oral anticoagulation with phenprocoumon in regular medical care and its potential for improvement in a telemedicine-based coagulation service – results from the prospective, multi-center, observational cohort study thrombEVAL

**DOI:** 10.1186/s12916-015-0268-9

**Published:** 2015-01-23

**Authors:** Jürgen H Prochaska, Sebastian Göbel, Karsten Keller, Meike Coldewey, Alexander Ullmann, Heidrun Lamparter, Claus Jünger, Zaid Al-Bayati, Christina Baer, Ulrich Walter, Christoph Bickel, Hugo ten Cate, Thomas Münzel, Philipp S Wild

**Affiliations:** Center for Thrombosis and Hemostasis, University Medical Center Mainz, Johannes Gutenberg University Mainz, Langenbeckstr. 1, 55131 Mainz, Germany; 2. Medizinische Klinik und Poliklinik, University Medical Center Mainz, Johannes Gutenberg University Mainz, Langenbeckstr. 1, 55131 Mainz, Germany; German Center for Cardiovascular Research (DZHK), Partner Site RheinMain, Langenbeckstr. 1, 55131 Mainz, Germany; Department of Medicine I, Federal Armed Forces Central Hospital Koblenz, Rübenacher Str. 170, 56072 Koblenz, Germany; Thrombosis Center Maastricht, Cardiovascular Research Institute Maastricht and Maastricht University Medical Center, PO Box 616, 6200 MD Maastricht, the Netherlands; Preventive Cardiology and Preventive Medicine, 2. Medizinische Klinik und Poliklinik, University Medical Center Mainz, Johannes Gutenberg University Mainz, Langenbeckstr. 1, 55131 Mainz, Germany

**Keywords:** Coagulation service, Epidemiology, Health care research, Oral anticoagulation, Quality of therapy, Telemedicine

## Abstract

**Background:**

The majority of studies on quality of oral anticoagulation (OAC) therapy with vitamin K-antagonists are performed with short-acting warfarin. Data on long-acting phenprocoumon, which is frequently used in Europe for OAC therapy and is considered to enable more stable therapy adjustment, are scarce. In this study, we aimed to assess quality of OAC therapy with phenprocoumon in regular medical care and to evaluate its potential for optimization in a telemedicine-based coagulation service.

**Methods:**

In the prospective observational cohort study program thrombEVAL we investigated 2,011 patients from regular medical care in a multi-center cohort study and 760 patients from a telemedicine-based coagulation service in a single-center cohort study. Data were obtained from self-reported data, computer-assisted personal interviews, and laboratory measurements according to standard operating procedures with detailed quality control. Time in therapeutic range (TTR) was calculated by linear interpolation method to assess quality of OAC therapy. Study monitoring was carried out by an independent institution.

**Results:**

Overall, 15,377 treatment years and 48,955 international normalized ratio (INR) measurements were analyzed. Quality of anticoagulation, as measured by median TTR, was 66.3% (inte rquartile range (IQR) 47.8/81.9) in regular medical care and 75.5% (IQR 64.2/84.4) in the coagulation service (*P* <0.001). Stable anticoagulation control within therapeutic range was achieved in 63.8% of patients in regular medical care with TTR at 72.1% (IQR 58.3/84.7) as compared to 96.4% of patients in the coagulation service with TTR at 76.2% [(IQR 65.6/84.7); *P* = 0.001)]. Prospective follow-up of coagulation service patients with pretreatment in regular medical care showed an improvement of the TTR from 66.2% (IQR 49.0/83.6) to 74.5% (IQR 62.9/84.2; *P* <0.0001) in the coagulation service. Treatment in the coagulation service contributed to an optimization of the profile of time outside therapeutic range, a 2.2-fold increase of stabile INR adjustment and a significant decrease in TTR variability by 36% (*P* <0.001).

**Conclusions:**

Quality of anticoagulation with phenprocoumon was comparably high in this real-world sample of regular medical care. Treatment in a telemedicine-based coagulation service substantially improved quality of OAC therapy with regard to TTR level, frequency of stable anticoagulation control, and TTR variability.

**Trial registration:**

ClinicalTrials.gov, unique identifier NCT01809015, March 8, 2013.

**Electronic supplementary material:**

The online version of this article (doi:10.1186/s12916-015-0268-9) contains supplementary material, which is available to authorized users.

## Background

Oral anticoagulation (OAC) therapy is the established therapy to reduce the risk of thromboembolic events in patients with atrial fibrillation [1]. Furthermore, it is applied for secondary prevention in patients with thrombosis, pulmonary embolism, and prosthetic heart valves [2]. In the future, the increasing age of the population will lead to a higher prevalence of atrial fibrillation, the most frequent indication for OAC therapy, and this will strongly increase the need for long-term treatment with oral anticoagulants [3]. Vitamin K antagonists (VKAs) have been demonstrated to be effective oral anticoagulant agents in reducing the risk of thromboembolic events [4]. OAC therapy with VKAs has to be regularly monitored and adjusted in order to maintain patients’ international normalized ratio (INR) levels within target range and, consequently, to minimize adverse events. The benefits of OAC therapy highly depend on quality of treatment as measured by time in therapeutic range (TTR) [5,6]. Calculation of TTR is an established surrogate parameter to assess quality of anticoagulation treatment with VKA [7] and is closely correlated with outcome and frequency of thromboembolic events [5,8]. In well-controlled and monitored clinical trials, TTR of short-acting warfarin ranged from 55% to 67% [6,9-11]. In contrast, levels of TTR of VKA treatment in real world settings have been reported to be substantially lower [12,13].

Different approaches have been made to optimize the management of VKA-based OAC therapy ranging from community-based settings to specialized care at anticoagulation clinics and self-management education of patients. Anticoagulation clinics introduced in the Netherlands have been demonstrated to be associated with higher levels of TTR [14] as compared to data from regular medical care as well as to operate cost-effectively [15]. The choice of VKA – either short-acting (e.g., acenocoumarol or warfarin) or long-acting (e.g., phenprocoumon) – seems to be also of importance due to inherent differences of substances regarding pharmacodynamics and pharmacokinetics [16]. In the Netherlands, where anticoagulation clinics provide the management of OAC therapy nationwide, it has been demonstrated that quality of therapy with long-acting phenprocoumon is superior to short-acting acenocoumarol [17]. Worldwide, short-acting warfarin is the most commonly prescribed oral anticoagulant drug and has been studied intensively [18]. Nevertheless, long-acting phenprocoumon is also widely administered as oral anticoagulant agent [19]. Although a class-effect for all VKA substances regarding quality of therapy and clinical outcome has been postulated, evidence from large-scale studies on quality of OAC therapy with phenprocoumon reporting on real-life practice (comprising all indications for OAC), clinical outcome, and the potential for optimization of OAC therapy by a coagulation service are, to the best of our knowledge, currently not available in the literature.

The thrombEVAL study program represents a prospective, multicenter observational study program which was initiated in 2011 to comprehensively investigate OAC therapy with phenprocoumon in the real-life setting. This study took place against a background of increasing application of new direct oral anticoagulants (currently known as non-vitamin K dependent oral anticoagulants, or NOACs [20]). This class of NOACs have been shown to be effective and relatively safe as compared to warfarin for several indications in large clinical trials. Although without doubt the NOACs will replace a major fraction of OAC treatment in patients with atrial fibrillation and venous thromboembolism, a significant proportion of these patients cannot yet be treated with these drugs. Reasons for choosing conventional VKA are severely impaired renal function, expected lack of adherence to medication that lacks routine control, and other concerns based largely on exclusion criteria applied in the large NOAC trials. In addition, patients with specific indications for OAC, including mechanical heart valves, cannot yet be treated with NOACs and it is uncertain whether this will ever be possible. Thus, worldwide, there will remain an indication for the use of well-controlled VKA, also in the long run. In addition, it is questionable whether the NOACs can really do without monitoring and dose adjustment, which is one of the proposed advantages of these agents. This advantage has now been called into question because recent reports indicate that adjustments of dabigatran dose in response to measurements of plasma concentrations may have little impact on stroke, but would strongly reduce the likelihood of major bleeds. These new results may indicate that, in case of a high TTR, VKAs still may represent a cheap and reliable alternative to NOACs for anticoagulation patients with, for example, atrial fibrillation [21,22].

Herein, the quality of OAC treatment as assessed by TTR in a population predominantly treated with phenprocoumon in regular medical care is reported. In order to evaluate the potential for improvement of OAC therapy with phenprocoumon, a specialized, telemedicine-based coagulation service was comparatively investigated.

## Methods

### Study design

The background and design of the thrombEVAL study program has been recently published elsewhere [23]. Briefly, it comprises two observational prospective studies which are performed within the German Health care system to investigate OAC treatment in a population which is predominantly treated with phenprocoumon: a multi-center cohort study with 21 study centers in regular medical care and a single-center, multi-local cohort study in a specialized, telemedicine-based coagulation service. Recruitment of study participants for both cohorts was performed between January 2011 and March 2013. Recruitment of patients for observational investigation in the thrombEVAL study did not influence management of OAC; prescription of drugs and dosing was independently performed by GP and ambulatory working specialists. The final sample size of the regular medical care cohort comprised 2,011 study participants, whereas 760 study participants were enrolled into the coagulation service cohort [23].

Both cohorts received detailed clinical assessment at study inclusion. In regular medical care, anticoagulation control was recorded from documentation of OAC therapy (e.g., anticoagulation pass). In the coagulation service, anticoagulation control was permanently documented in an electronic patient file for up to 2 years. To depict a real-life scenario of anticoagulation practice, patients with all indications for OAC therapy were eligible for study enrolment. The trial was designed and led by a steering committee of academic investigators. The study coordination, management of database, and primary analysis were independently performed by the Center for Thrombosis and Hemostasis (Mainz, Germany). Study monitoring was carried out by an independent institution; all procedures were performed according to the principles of good clinical practice, STROBE guidelines, and the Declaration of Helsinki. Approval of the local ethics committees (medical association Rhine-Hesse, Germany; reference no. 837.407.10.7415/7416) was obtained at all sites.

### Study participants

Patients were recruited from a mid-western population of predominant white European ancestry. In regular medical care, patients were eligible if OAC experience of at least 4 months duration was present in patients before study enrolment. For the coagulation service cohort, VKA-naive and VKA-experienced patients with envisaged treatment duration of at least 3 months were eligible for study enrolment. Reasons for exclusion were age <18 years and contraindication to OAC treatment, e.g., pregnancy. Patients performing self-management of OAC (including point-of-care blood withdrawal and VKA self-dosing) were eligible for both cohorts. As this was an observational study, it did not interfere with any other medical treatment. All study participants provided written, informed consent.

### Data assessment and study procedures

After study enrolment all study participants underwent baseline investigation including clinical data assessment, characteristics of OAC treatment, and acquisition of history of OAC (e.g., retrospective analysis of documentation of anticoagulation therapy). All data were obtained according to standard operating procedures. Data assessment was performed via structured computer-guided analysis with checks for plausibility and validity.

At the coagulation service, OAC therapy was monitored by nurses with training in hemostaseology and experienced physicians. All treatment information was integrated into an electronic patient file, which could be accessed via secured internet-connection and enabled telemedicine-based bridging of spatial and temporal distances between patients, the coagulation service, and other physicians in charge. Anticoagulant dose-adjustment was based on the use of electronic patient file data and integrated computer-assisted dosing algorithms. Automated scheduling of OAC control visits was established to prevent loss to follow-up and improve patient adherence.

In the coagulation service, patients with self-management of OAC had the possibility to enter INR values and dosing schemes in the electronic patient file. All entries to the file were inspected by the staff and, if necessary or requested (e.g., problems with dosing or INR measurement, management of bridging episodes), medical advice was provided. Patients with self-management in regular medical care performed INR measurements and adjustment of VKA dosing independently.

INR values were obtained by analysis of anticoagulation documentation (regular medical care cohort) and electronic patient files (coagulation service cohort). Double data entry of all paper-based documents into the study database ensured high quality.

### Statistical analysis

In this analysis, data on the primary short-term outcome of the thrombEVAL study program, TTR, is reported. Calculation of TTR was performed by the linear interpolation method [7]. In order to enable comparative analysis between the cohorts, TTR was calculated only in patients with a consecutive lifetime use of VKA of at least 4 months. Patients with self-management of OAC were analyzed separately. Specific target ranges were taken into account according to indication for OAC. INR values were taken into account for TTR calculation from the moment that the first INR value was within therapeutic range. Assessment of INR values identifying patients with stable anticoagulation control was carried out separately. In order to enable comparability between the cohorts, patients with self-management of OAC, or pretreatment with OCA <4 months, or treatment in a specialized outpatient clinic were analyzed separately from overall comparison of standard care in both cohorts. Classification of “patient with stable anticoagulation control” (within individual therapeutic target range) required VKA treatment of at least 28 days with three consecutive INR measurements within therapeutic range. According to the interpolation method for calculating time in therapeutic range, the profile of time outside therapeutic range was analyzed. Time of INR values outside the target range was calculated and as was, subsequently, the distribution of time above and below therapeutic range. TTR and time outside therapeutic range were expressed as median values with 25^th^/75^th^ percentiles (interquartile range, IQR) or as mean values ± standard deviation, respectively. For TTR comparison between mixed samples of paired and unpaired data a corrected z-test was used. Variability of TTR profile and time outside therapeutic range between regular medical care and the coagulation service, respectively, were tested for by the one-sided Ansari-Bradley test, a test for equivalence of variances in two distributions [24]. Discrete variables were described by absolute and relative frequencies. Fisher’s exact test was used to test for proportion differences. The Mann-Whitney U-test was performed to test for difference of medians in the two groups; the z-test was used to test for difference of means between groups of paired and unpaired data. All statistical comparisons were two-tailed. *P* <0.05 was chosen as the statistical significance threshold. Statistical data analysis was performed using R version 2.14.1 (The R Project for Statistical Computing).

## Results

### Analysis key data

This analysis included the data of 2,771 patients that were enrolled between January 2011 and March 2013, including 2,011 participants in regular medical care and 760 in the coagulation service. A total of 15,377 treatment years and 48,955 INR measurements were available for analysis. For cross-sectional analysis, information on regular medical care pretreatment data of coagulation service patients was assigned to the regular medical care cohort.

Anticoagulation pass was in use in 1,924 of 2,011 patients (95.7%) of regular medical care participants and available for analysis in 1,774 of 1,924 patients (92.2%). After analysis of OAC documentation calculation of TTR according to linear interpolation method was applicable in 1,348 out of 1,774 patients of regular medical care (76.0%) based upon anticoagulation pass documentation including 188 participants performing self-management of OAC. For overall comparison of standard care in both cohorts, 1,160 patients of the regular medical care cohort were finally eligible. In the coagulation service, electronic patient file data were available in all 760 (100%) patients. Calculation of TTR was applicable in 723 of 760 (95.1%) coagulation service patients, including 72 of patients with self-management associated to the coagulation service. In 560 out of 723 participants (77.5%), regular medical care inclusion criteria were applicable in order to perform analysis of both samples under equal assumptions.

### Patient characteristics

The characteristics of the study participants are shown in Table 1. Median age showed no differences between regular medical care and the coagulation service. Both cohorts showed a high cardiovascular risk profile; hypertension was the most common risk factor in both cohorts. Treatment characteristics of the study participants are displayed in Table 2. Phenprocoumon was the VKA of choice in approximately 98% of all patients in both cohorts.

**Table 1:** **Baseline characteristics of study participants**

**Characteristic**	**Regular medical care**	**Coagulation service**
Subjects	2,011	760
Male sex, % (no.)	62.2 (1,251)	52.0 (395)
Age, years	73.0 (66.0/79.0)	73.0 (63.0/80.0)
Body mass index, kg/m^2^	27.6 (24.7/31.1)	27.8 (24.9/31.2)
**Classical cardiovascular risk factors**		
Diabetes, % (no.)	30.8 (617)	26.0 (196)
Dyslipidemia, % (no.)	51.9 (1,043)	42.0 (319)
Family history of MI and/or stroke/TIA, % (no.)	38.2 (768)	30.4 (231)
Hypertension, % (no.)	79.1 (1,590)	75.7 (575)
Obesity, % (no.)	30.6 (616)	31.6 (240)
Smoking, % (no.)	6.7 (135)	5.5 (42)
**Concomitant diseases**		
Atrial fibrillation, % (no.)	72.7 (1,452)	63.6 (483)
Autoimmune disease, % (no.)	8.6 (170)	7.4 (56)
Chronic kidney disease, % (no.)	22.4 (447)	15.8 (120)
Chronic obstructive pulmonary disease, % (no.)	21.0 (417)	15.6 (118)
Coronary artery disease, % (no.)	39.9 (773)	27.8 (210)
Depression, % (no.)	8.8 (176)	7.7 (58)
Heart failure, % (no.)	41.2 (813)	30.5 (230)
Liver disease, % (no.)	5.6 (112)	3.4 (26)
Myocardial infarction, % (no.)	20.0 (400)	12.2 (92)
Neoplasm, % (no.)	17.8 (354)	19.0 (142)
Peripheral artery disease, % (no.)	20.7 (408)	11.1 (84)
Sleep apnea, % (no.)	9.7 (186)	7.8 (58)
Stroke or TIA, % (no.)	17.3 (348)	17.5 (133)

Data are expressed as the relative and absolute frequencies for binary variables, for normally distributed variables as median with 25^th^/75^th^ percentile. Double entries are possible for study participants in the coagulation service cohort with prior treatment in regular medical care. TIA, Transient ischemic attack; MI, Myocardial infarction. Significant difference between the groups (*P* <0.05) was detected for hypertension, dyslipidemia, family history of MI/stroke/TIA, coronary artery disease, myocardial infarction, heart failure, peripheral artery disease, atrial fibrillation, chronic kidney disease, and liver disease.

**Table 2:** **Treatment characteristics of study participants**

**Characteristic**	**Regular medical care**	**Coagulation service**
Total amount of treatment days	4,681,125	931,579
Total amount of international normalized ratio (INR) values	29,748	19,207
Median time between INR measurements [days; interquartile range]	17.0 (10.7/26.0)	15.2 (11.4/18.8)
Self-management of oral anticoagulant therapy	13.5 (271)	9.5 (72)
Physician in charge		
General practitioner, % (no.)	67.6 (1,359)	n.a.*
Specialist, % (no.)	32.3 (650)	n.a.*
Home visits, % (no.)	6.8 (137)	8.8 (67)
Vitamin K antagonist in use		
Warfarin, % (no.)	1.7 (34)	1.7 (13)
Phenprocoumon, % (no.)	98.3 (1,977)	98.3 (747)

Data are expressed as the relative and absolute frequencies for binary variables. *Due to management of anticoagulation in the coagulation service classification of “physician in charge” was not applicable in these patients.

### Quality of oral anticoagulation therapy

**Figure 1:**
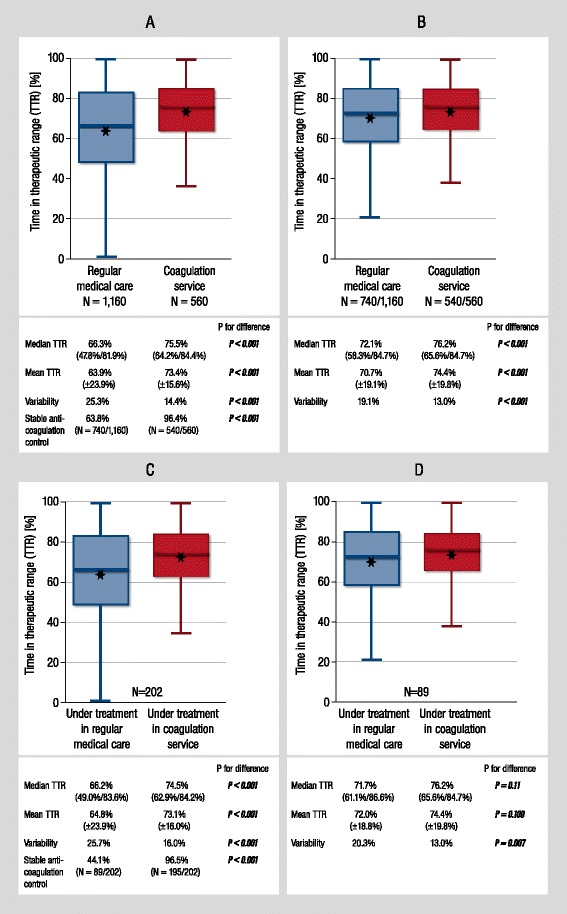
**Quality of oral anticoagulation therapy in regular medical care and a telemedicine-based coagulation service.** **(A)** Comparison of quality of oral anticoagulation therapy in in patients of regular medical care and coagulation service. **(B)** Comparison of quality of oral anticoagulation therapy in in patients of regular medical care and coagulation service in subsample of patients with stable anticoagulation control. **(C)** Intra-individual comparison of quality of oral anticoagulation in patients treated first in regular medical care (blue) and afterwards in coagulation service (red). **(D)** Intra-individual comparison of quality of oral anticoagulation in patients treated first in regular medical care (blue) and afterwards in coagulation service (red) in subsample of patients with stable anticoagulation control. Time in therapeutic range is calculated according to linear interpolation method and presented as median (first quartile/third quartile); *P* value for z-test. Mean TTR values are depicted graphically as asterisks within box-plots. TTR variability is expressed by median absolute deviation, *P* value for Ansari-Bradley test. Absolute and relative frequency of stable oral anticoagulation control is depicted.

In comparison to regular medical care, median TTR was 9.2% higher in the coagulation service cohort (75.5% (64.2/84.4) vs. 66.3% (47.8/81.9); *P* <0.001). Stable anticoagulation control was 1.5-fold more frequent in coagulation service patients as compared to regular medical care patients (96.4% vs. 63.8%; *P* <0.001) and variability of TTR was reduced by 43.0% in coagulation service patients (14.4 vs. 25.2; *P* <0.001; Figure 1A). Sub-analysis of patients with stable anticoagulation control revealed similar findings: TTR was at higher level in the coagulation service cohort (76.2% (65.6/84.7) vs. 72.1% (58.3/84.7); *P* <0.001) and variability of TTR was 31.9% lower in coagulation service patients (13.0 vs. 19.1; *P* <0.001; Figure 1B).

In coagulation service patients with pretreatment in regular medical care, level of TTR at time of regular medical care treatment was not statistically different from the TTR of the regular medical care cohort (66.2% vs. 68.5%; *P* = 0.72). Prospective follow-up during treatment in the coagulation service revealed a highly significant increase of TTR upon coagulation service treatment (66.2% (49.0/83.6) to 74.5% (62.9/84.2; *P* <0.001; Figure 1C). In patients with stable anticoagulation control in regular medical care, additional improvement of median TTR of 5.9% (77.6% (66.3/85.5) vs. 71.7% (61.1/86.6); *P* <0.001) was detected together with a decrease of TTR variability by 36% (20.3 vs. 13.0; *P* = 0.009). Frequency of stable anticoagulation control was 2.2-fold greater after coagulation service treatment as compared to regular medical care pretreatment (96.5% vs. 44.1%; *P* <0.001; Figure 1D). Quality of OAC therapy for the subsample of patients treated with phenprocoumon did not differ from quality of the overall sample of all VKA-treated patients (see Additional file 1: Table S1).

### Indications for oral anticoagulation (OAC) therapy

Atrial fibrillation was the most frequent indication for OAC in both cohorts. In comparison to venous thromboembolism, which was the second frequent indication for OAC, atrial fibrillation was 4.7-fold (regular medical care) and 2.3-fold (coagulation service) more prevalent, respectively. Quality of OAC differed between indications and cohorts: in regular medical care, patients with prosthetic heart valves showed the poorest anticoagulation control whereas patients with atrial fibrillation achieved the highest level of TTR. In the coagulation service, distribution of TTR among all indications for OAC presented a consistent and ameliorated profile of TTR (except for other indications). Patients with a prosthetic heart valve showed the most distinct difference in quality of therapy between both cohorts with a ∆TTR of +24.6% in the coagulation service cohort (Table 3).

**Table 3:** **Distribution of indication for oral anticoagulation (OAC) and corresponding time in therapeutic range in regular medical care and coagulation service**

**Indication for OAC**	**Regular medical care**		**Coagulation service**	
	**Frequency**	**Time in therapeutic range**	**Frequency**	**Time in therapeutic range**
Atrial fibrillation	66.2% (1,332)	67.5% (49.3/83.3)	61.1% (464)	75.0% (61.8/77.3)
Deep vein thrombosis	6.1% (123)	65.2% (46.8/75.3)	14.2% (108)	75.3% (66.1/85.0)
Peripheral vascular bypass surgery	8.0% (160)	64.9% (47.1/81.2)	2.2% (17)	74.9% (61.8/77.3)
Prosthetic heart valve	9.7% (195)	42.2% (30.4/68.3)	7.4% (56)	76.8% (63.0/82.8)
Pulmonary embolism	7.7% (154)	66.5% (50.6/82.6)	13.3% (101)	75.5% (64.7/84.7)
Others*	5.0% (100)	70.1% (54.3/82.1)	5.0% (38)	79.2% (59.8/88.0)

Patients can have more than one indication for oral anticoagulation (OAC) with vitamin K antagonist; indication is described in 2,011 of patients in regular medical care and 760 patients in the coagulation service cohort. In coagulation service patients with pre-treatment in regular medical care, information on regular medical care pre-treatment are described within regular medical care cohort (non-disjunct data). Frequency of indication is depicted as relative and absolute frequency. Time in therapeutic range was calculated in patients with at least 4 months of anticoagulation treatment except self-management patients (1,160 patients in regular medical care and 560 patients in coagulation service, respectively). *e.g., cerebral venous sinus thrombosis, Paget-Schrötter disease.

### Profile of time outside therapeutic range

**Figure 2:**
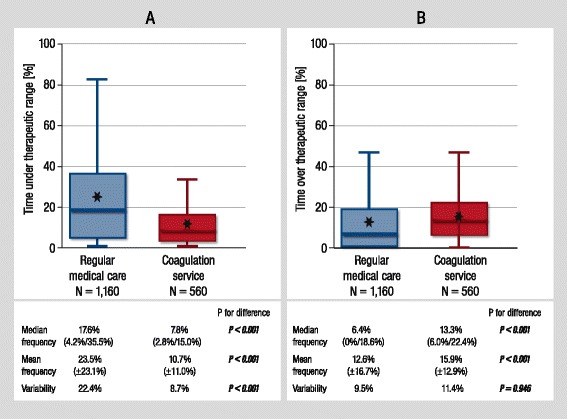
**Profile of time outside therapeutic range in regular medical care and coagulation service.** **(A)** Relative frequency of time below therapeutic range. **(B) **Relative frequency of time above therapeutic range. Box-plots of profile of time outside therapeutic range of regular medical care and coagulation service. Time outside therapeutic range is presented as median (first quartile/third quartile); mean values are depicted graphically as asterisks within box-plots. Variability of frequency outside therapeutic range is expressed by median absolute deviation, *P* value for Ansari-Bradley test.

The profile of the time outside therapeutic range in both cohorts is depicted in Figure 2. In the regular medical care cohort, the profile of time outside therapeutic range presented an approximately 3-fold higher level of under-anticoagulation (Figure 2A) as compared to over-anticoagulation (Figure 2B; median frequency of under-anticoagulation: 17.6% (4.2/35.5), median frequency of over-anticoagulation: 6.4% (0/19.6)). In coagulation service patients, the profile showed a more leveled balance between over- and under-anticoagulation: the ratio of under-treatment to over-treatment was 2.8 in regular medical care and 0.6 in coagulation service patients (4.7-fold reduction). The level of INR values below TTR was reduced by 44.3% (*P* <0.001); in addition, variability of values below TTR was 2.6-fold higher in regular medical care (*P* <0.0001). Levels of INR above the TTR did not differ statistically between both cohorts (*P* = 0.946).

### Patients with self-management of oral anticoagulation (OAC) therapy

In patients performing self-management of OAC, TTR was detected to be at a higher level of quality in comparison to physician-guided OAC in both regular medical care and coagulation service treatment. In these patients, a high quality of therapy is obtained at the price of an approximately doubled frequency of INR control as compared to regular patients. Overall, levels of median TTR were higher for patients with self-management of OAC who were affiliated to a coagulation service as compared to regular medical care patients, although it did not reach a level of statistical significance (84.8% (49.0/83.6) vs. 86.0% (62.9/84.2; *P* = 0.075). In self-management patients associated to the coagulation service, variability of TTR was 19.7% significantly lower (14.7 vs. 18.3; *P =* 0.031) and stable anticoagulation control was obtained 13.7% more frequently in these patients (93.6% vs. 80.9%; Additional file 2: Figure S1).

### Time-dependent development of TTR

**Figure 3:**
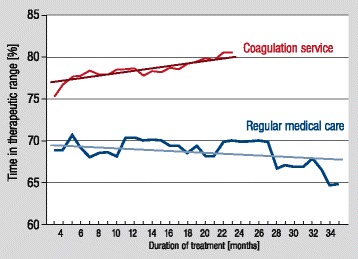
**Development of time in therapeutic range over time in a specialized coagulation service in comparison to regular medical care.** Time in therapeutic range (TTR) is assessed according to linear interpolation method. Median TTR values are depicted for both cohorts for each time point. Values of regular medical care are demonstrated in red, for coagulation service in blue.

In both cohorts, development of TTR over time differed significantly: in regular medical care, a decrease of TTR of 0.053% per month was observed for the maximum observation period of 35 months. In comparison, in the coagulation service, an increase of 0.15% per treatment months was detected (Figure 3). Besides a difference of approximately 5.2% ∆TTR after 3 months of oral anticoagulants, an additional difference of 3.0% in TTR was discovered after 12 months of treatment in coagulation service patients (*P* for interaction <0.0001).

## Discussion

In this study, a surprisingly high level of TTR in a population predominantly treated with the long-acting VKA phenprocoumon in a real-world setting of regular medical care was obtained. Management of OAC therapy by a specialized, telemedicine-based coagulation service was able to substantially improve the quality of anticoagulation therapy: higher levels of TTR were established for all patients and for all indications. In addition, an increase in the frequency of stable anticoagulation control and a decrease of variability of TTR was observed.

The finding of high levels of TTR in this cohort with phenprocoumon-based OAC in regular medical care might be – at least in part – attributable to the use of phenprocoumon as a long-acting VKA. In comparison to short-acting acenocoumarol, phenprocoumon has been reported to be associated with higher levels of TTR in the Netherlands [25]. This finding is of clinical relevance since most evidence on OAC is derived from data on well-investigated warfarin and extrapolated to other VKA. Median TTR in warfarin-driven, controlled clinical trials, in which detailed control of therapy adherence was carried out, was reported to be below 67% [26-28]. For community practice, significantly worse quality of anticoagulation control has been demonstrated in comparison to clinical trials [9]: a meta-analysis of 8 studies with a total of 22,237 warfarin-treated patients reported TTR to be at 51% in community practice under warfarin treatment. Differences in pharmacodynamics between short- and long-acting VKA drugs may contribute to differences in variation of INR under therapy in favor of the longer acting agents. This may indeed enable more stable anticoagulation control with phenprocoumon as compared to the shorter acting products. As previously described, patients on phenprocoumon seem to require fewer monitoring visits and have more stable INR values than patients treated with short-acting VKA [29]. In addition, effects of VKA-relevant genetic polymorphisms, e.g., CYP2C9 or VKORC-1 polymorphism [30,31], might be less pronounced in patients treated with phenprocoumon [16]. Interestingly, in the present analysis, the majority of patients – especially in the coagulation service – presented stable anticoagulation control with higher than average levels of TTR control. Against the background that combined rates of bleeding and thromboembolism are significantly lower in stable patients [32], these data indicate that phenprocoumon-driven OAC may provide high quality therapy, even in a non-specialized setting.

Differences in TTR between indication groups revealed that, especially in patients with prosthetic heart valves who are treated in regular medical care, the quality of OAC was the worst in this patient collective in regular medical care. Patients with these indications for anticoagulation are known to have the highest risk for life-threatening bleeding and thromboembolic events. Hence, the significant increase of TTR in the coagulation service seems highly clinically relevant. This is even more important as application of NOACs for the indication of mechanical heart valves is no valid option as yet, as illustrated by the data of the recent RE-ALIGN study demonstrating that the application of the novel direct anticoagulant dabigatran was associated with increased rates of thromboembolic and bleeding complications as compared to standard VKA therapy [33]. Self-management of OAC therapy is often performed by these patients, and has been demonstrated to yield good quality of therapy control. This finding is in line with a randomized controlled trial on selected patients, which demonstrated that the quality of patient self-management of OAC is comparable to specialized anticoagulation clinics, at least for a selected group of patients [25,34].

The finding that a high level of quality of OAC can be achieved by a specialized coagulation service for all groups of patients is in line with results from other European anticoagulation clinics: data from the AuriculA investigators of the Swedish national quality registry for anticoagulation and atrial fibrillation control reported a mean TTR of 76.2% for the participating anticoagulation centers in Sweden [14]. Management of OAC by anticoagulation clinics appears to generate less costs and provide greater effectiveness than usual care [15]. These data are confirmed by reports from the Netherlands where anticoagulation clinics achieve high-quality OAC with low rates of adverse events [35]. Based on the strong relation between time spent in therapeutic range and clinical outcome [36-38], the reported difference in TTR between regular medical care and treatment in a coagulation service in the present study is likely to be translated into a reduction of adverse events [8,39]. A sub-analysis of the RE-LY study stressed the interdependence of quality of INR control and clinical outcome: although non-inferiority of dabigatran was demonstrated to be presented at different levels of TTR control, high quality VKA treatment at TTR >72.4% was shown to cause fewer ischemic and hemorrhagic strokes. Depending on TTR, dabigatran at a dose of 150 mg lost superiority over warfarin in high TTR study centers regarding the reduction of the risk of non-hemorrhagic stroke [40]. Optimization of the profile of time outside therapeutic range, increase of frequency of stable anticoagulation control, and less TTR variability may additionally contribute to a reduction of thromboembolic and bleeding events in coagulation service patients [41]. However, prospective data on clinical outcome of patients in both cohorts are necessary to validate this intriguing finding.

In the coagulation service, which offers a multi-factorial approach for comprehensive management of OAC therapy, various aspects of the complexity of OAC have been addressed, e.g., algorithm-driven phenprocoumon dosing with respect to individual information in context to the visit, patient education, and minimization of gaps in OAC monitoring [42]. Key challenges, such as adherence to therapy and medication, use of standard INR ranges, and optimal treatment of comorbidities, have to be addressed in order to obtain high quality of OAC. It seems elementary that any decision about VKA dose changes must occur in the context of the anticoagulation visit and reported patient information, also when using dosing algorithms for TTR improvement. In the context of demographic changes with an increasing amount of multi-morbid patients and a still high level of OAC under-use, especially in the elderly, future concepts for OAC will have to address a variety of issues in a multimodal approach [3,43,44]. Therefore, a specialized coagulation service provides the unique potential to combine high quality OAC together with continuous scientific evaluation; this may be especially of importance for future considerations on the management of OAC, also with respect to the introduction of new OACs into clinical practice and an emerging necessity for an evidence-based and individually-tailored OAC therapy.

### Strength and limitations

A major strength of the present study is the large-scale investigation of quality of OAC therapy predominantly performed with phenprocoumon in a real-life setting of regular medical care and evaluation of its potential for improvement in a specialized coagulation service. In contrast to most reports in the literature, all indications for OAC are included in the present investigation. However, the present analysis has several limitations. In this analysis, calculation of the established surrogate parameter TTR is performed to compare quality of OAC; although it is well-recognized that efficacy and safety of OAC with VKA is TTR-dependent [45], potential differences in clinical outcome between patients treated in regular medical care and a coagulation service can only be estimated. Extrapolation of TTR values of a sample of predominantly white and European ancestry to other populations should be done with caution. TTR was calculated according to the widely used interpolation method [7], nevertheless, gaps in INR monitoring in the regular medical care cohort, which have not been documented, cannot be ruled out. For methodological reasons, extreme ranges of INR values may bias overall results. It was not analyzed how improvement of TTR was achieved by the coagulation service. The potential of dosing algorithms to improve TTR has been described in several studies [46-48]. Of course, there may be other computer-assisted dosing algorithms than the one investigated in the thrombEVAL study program that result in even better INR adjustment. In regular medical care, only patients with a minimum experience of 4 months of OAC have been enrolled; therefore, development of TTR after therapy initiation is not included in this analysis. Due to the nature of an observational “real-world” study, selection and survival bias may limit the extrapolation of the study results.

## Conclusions

The key result of the current study is the demonstration of an excellent high level of TTR using the long-acting phenprocoumon in a telemedicine-based coagulation service. Treatment in a coagulation service with a standardized and multifactorial approach improved the quality of therapy for patients regardless of indication of OAC. This finding corroborates the importance of anticoagulation clinics, which are pivotal for good quality anticoagulation with VKAs, but which presumably could also play a role in the long-term management of NOACs. The latter is underscored by recent reports on the association between plasma levels of NOAC, in this case dabigatran, and clinical outcomes, suggesting that individual tailoring towards an optimal dose will be unavoidable [49-51]. For the timbering of patients, properly managed treatment with VKAs in a telemedicine-based setting seems a reasonable alternative to unmonitored NOAC treatment. In addition, monitoring of patients with NOAC in a telemedicine-based setting may be an option for safe guidance of OAC patients.

## Additional files

Additional file 1: Table S1. Quality of oral anticoagulation therapy in patients treated with phenprocoumon only. Treatment with phenprocoumon was present in 1,977 of 2,011 patients in regular medical care and 723 of 760 patients in coagulation service. Calculation of TTR calculation was applicable in 1,143 patients of regular medical care and 614 patients of coagulation service. Data are expressed for variable TTR as median (first quartile/third quartile). Additional file 2: Figure S1. Quality of oral anticoagulation therapy of patients with self-management in regular medical care and coagulation service. **(A)** Comparison of patients with self-management of oral anticoagulation in regular medical care and coagulation service. **(B)** Subsample of self-management patients with stable anticoagulation control. Time in therapeutic range is calculated according to linear interpolation method and presented as median (first quartile/third quartile); *P* value for z-test. Mean TTR values are depicted graphically as asterisks within box-plots. *TTR variability is expressed by median absolute deviation, *P* value for Ansari-Bradley test. Absolute and relative frequency of stable oral anticoagulation control is depicted.

## Abbreviations

INR: International normalized ratio
IQR: Interquartile range, NOAC, Non-vitamin K dependent oral anticoagulant drug
OAC: Oral anticoagulation
TTR: Time in therapeutic range
VKA: Vitamin K-antagonists
