# Supplementary material for: Quality of oral anticoagulation with phenprocoumon in regular medical care and its potential for improvement in a telemedicine-based coagulation service – results from the prospective, multi-center, observational cohort study thrombEVAL
Source: BMC Med. 2015 Jan 23;13:14. doi: 10.1186/s12916-015-0268-9 (PMC4333875; doi:10.1186/s12916-015-0268-9)
Supplement: Additional file 1: Table S1. — Quality of oral anticoagulation therapy in patients treated with phenprocoumon only. Treatment with phenprocoumon was present in 1,977 of 2,011 patients in regular medical care and 723 of 760 patients in coagulation service. Calculation of TTR calculation was applicable in 1,143 patients of regular medical care and 614 patients of coagulation service. Data are expressed for variable TTR as median (first quartile/third quartile). [file 12916_2015_268_MOESM1_ESM.doc]

Additional file 1

Table S1. Quality of Oral Anticoagulation Therapy in Patients treated with Phenprocoumon only

|  | **Time in therapeutic range (TTR)** | **Time below therapeutic range** | **Time above therapeutic range** |
| --- | --- | --- | --- |
| **Patients treated with phenprocoumon only** |  |  |  |
| Regular medical care | 66.5% (47.8/82.0) | 17.3% (4.1/35.4) | 6.5% (0/18.8) |
| Coagulation service | 75.7% (63.6/84.4) | 7.8% (2.8/15.0) | 13.3% (6.0/22.3) |

Treatment with phenprocoumon was present in 1,977 of 2,011 patients in RMC and 723 of 760 patients in CS. Calculation of TTR calculation was applicable in 1,143 patients of regular medical care and 614 patients of coagulation service. Data are expressed for variable TTR as median (first quartile/third quartile).
