# Supplementary material for: Quality of oral anticoagulation with phenprocoumon in regular medical care and its potential for improvement in a telemedicine-based coagulation service – results from the prospective, multi-center, observational cohort study thrombEVAL
Source: BMC Med. 2015 Jan 23;13:14. doi: 10.1186/s12916-015-0268-9 (PMC4333875; doi:10.1186/s12916-015-0268-9)
Supplement: Additional file 2: Figure S1. — Quality of oral anticoagulation therapy of patients with self-management in regular medical care and coagulation service. (A) Comparison of patients with self-management of oral anticoagulation in regular medical care and coagulation service. (B) Subsample of self-management patients with stable anticoagulation control. Time in therapeutic range is calculated according to linear interpolation method and presented as median (first quartile/third quartile); P value for z-test. Mean TTR values are depicted graphically as asterisks within box-plots. *TTR variability is expressed by median absolute deviation, P value for Ansari-Bradley test. Absolute and relative frequency of stable oral anticoagulation control is depicted. [file 12916_2015_268_MOESM2_ESM.doc]

**Additional file 2**

**Figure S1**

Title: Quality of Oral Anticoagulation Therapy of Patients with Self-Management in Regular Medical Care and Coagulation Service.

Caption: A. Comparison of Patients with Self-Management of oral Anticoagulation in Regular Medical Care and Coagulation Service. B. Subsample of Self-Management Patients with stable Anticoagulation Control. Time in therapeutic range is calculated according to linear interpolation method and presented as median (first quartile/third quartile); *P* value for z-test. Mean TTR values are depicted graphically as asterisks within box-plots. *TTR variability is expressed by median absolute deviation (MAD), *P* value for Ansari-Bradley-test. Absolute and relative frequency of stable oral Anticoagulation control is depicted.
